# Cybersecurity and the *Digital-Health*: The Challenge of This Millennium

**DOI:** 10.3390/healthcare9010062

**Published:** 2021-01-11

**Authors:** Daniele Giansanti

**Affiliations:** Centre Tisp, Istituto Superiore di Sanità, Via Regina Elena 299, 00161 Roma, Italy; daniele.giansanti@iss.it; Tel.: +39-06-4990-2701

## 1. Cybersecurity

The problem of computer security is as old as computers themselves and dates back decades. The transition from: (a) a single-user to multi-user assignment to the resource and (b) access to the computer resource of the standalone type to one of the types distributed through a network made it necessary to start talking about computer security. All network architectures, from *peer to peer* to *client-server* type, are subject to IT security problems.

The term *Cybersecurity* has recently been introduced to indicate the set of procedures and methodologies used to defend computers, servers, mobile devices, electronic systems, networks and data from malicious attacks. *Cybersecurity* [1,2,3] is therefore applied to various contexts, from the economic one to that relating to mobile technologies and includes various actions:

- *Network security*: the procedures for using the network safely;
- *Application Security*: the procedures and solutions for using applications safely;
- *Information security*: the management of information in a secure way and in a privacy-sensitive manner in accordance with pre-established regulations;
- *Operational security*: the security in IT operations, such as, for example, in bank-type transactions;
- *Disaster recovery and operational continuity*: the procedures for restarting after problems that have affected the regular/routine operation of a system and to ensure operational continuity. For example, using informatic solutions such as an efficient disk mirroring and/or backup policy;
- *End-user training*: specific training for the actors involved in the use of the systems, which where necessary, must also include the citizen.

## 2. Cybersecurity and Health Care

The recent decade has seen a growing interest in information security. *Cyber attacks* in the industry and consumer sectors have been widely echoed in the past and recent *cyber attacks* in the healthcare sector are of concern. Recently, for example, at the center of the debate were the attacks on health systems and the potential vulnerabilities that have come to light for some types of critical medical device (mostly active implantable) that can be connected to the network [1,2]. In several nations, there has generally been a delay in addressing *cybersecurity* issues compared to other nations, for example, the US. This is due to the fact that in the US, the world of health is undoubtedly an industry, not only in terms of perception, but in practice: the approach to the problem in the US has, in fact, been identical to that taken in general towards the world of industry and consumption. Only recently, however, has the problem begun to be given due attention. In the current healthcare sector, the criticality relating to the extraordinary diffusion of innovative technologies (e.g., artificial pancreas, pacemakers) connected to the network in the healthcare sector (over 300,000 classes of Medical Devices) are inevitably intertwined with the safety and efficacy characteristics of the services provided and the protection of the data processed, creating a context of high attention.

The *cybersecurity* in the healthcare:

- Includes all the general actions listed and described in the previous paragraph (Network Security, Application Security, Information Security, Operational Security, Disaster Recovery and Operational Continuity, End-User Training) tuned and specialized for the health-care sector;
- Faces *four main aspects* in the *cyber-system* that can either be a complex medical device (e.g., wearable pumps; wearable stimulators; pacemakers; artificial pancreas) and/or a complex interoperable and heterogeneous system (e.g., A Hospital Information System; a Radiology Information System; a dedicated medical network) comprehending several components of *elaboration systems, informatics, biomechatronics, bioengineering, electronics, networks*, *eHealth*, *mHealth* [1].

***The data preservation***

The procedures assuring the data for a prolonged period remains reachable and functioning. These procedures must respect adequate specifications and use informatic resources that are adequate for the purpose, such as adequate and stable filing systems.

***The data access and modification***

Refers to those typical functionalities such as storing and recovering data stored in databases or other archives. The implementation of these actions is obtained by means of specific designed procedures for the authentication and authorization of the regulated access. 

***The data exchange***

Data exchange can be carried out either internally (for example, in the Hospital LAN) or externally (from the Hospital Lan to the external actors, such as, for example, the citizen and/or other practitioners, and/or other healthcare bodies). It is evident that the data exchange should take in a safe way place, in compliance with defined security specifics, with the application of suitable measures of data protection. 

***The interoperability and compliance***

The *Interoperability* allows both a health-care worker and a citizen to exchange the data among several systems and devices in a shared manner. Two systems are considered interoperable when they are able to exchange data and later present that data so that they are comprehensible by all the involved actors. The *compliance refers* to the world of regulations. It deals, for example, both with the use of the same standards (e.g., Dicom in the radiology information systems) and with adherence to national and international regulations (e.g., the GPRS in Europe) concerning the usage of health information.

## 3. Specific Healthcare Sectors to Be Faced with Particular Attention in the Cybersecurity

### 3.1. Wearable Medical Device

The wearable medical devices [2,3,4,5,6], and, in particular, the implantable ones, are part of a heterogeneous system (e.g., pacemakers, artificial pancreas). In a heterogeneous system, the wireless connection allows the components to communicate with each other, and creates an environment potentially susceptible to *cyber attacks*. If the connection between the *wearable device* for continuous monitoring and the external elaborator is potentially unsafe, an attacker could send deliberately incorrect data to the control algorithm. For the *artificial pancreas,* for example, this could cause the release of a high amount of insulin, resulting in a situation of hypoglycemia in the patient; the body would respond to a hypoglycemic situation through the release of glucagon and epinephrine and continuing the situation would compromise the brain, motor and cognitive functions, even leading to death. For the *pacemaker,* this could cause the generation of an incorrect electronic pulse activity and create, for example, the dangerous fibrillations, which could rapidly lead to the death of the subject wearing the device. To take account of these issues, the Food and Drug Administration (FDA), for example, has made guidelines and recommendations available online.

### 3.2. Picture Archiving and Communication System

The *Picture Archiving and Communication System* (PACS) [2,3] is a medical device software (defined by the FDA as a Class II medical device) dedicated to the management of a diagnosis reached using the medical imaging. A PACS embeds several parts such as *elaborators, workstations, digital-databases, digital data-stores, digital-applications*. In the PACS, several software components are dedicated to the image downloading, uploading and manipulation. These actions clearly imply issues of data security and integrity, if we consider that a PACS is a deposit of patients’ data with the inference of aspects related to the *data privacy and protection*. It is evident that the *cybersecurity* assumes strategic importance in the PACS in several tasks/activities of the digital radiology, in particular:

1. During the diagnostic/decision-making processes;
2. During the various phases of information manipulation ranging from image acquisition to its storage and subsequent sharing according to *client/server* type architectures.

### 3.3. Health Care Networks

As is well known, hospital companies today are strongly based on digital technologies. The *cyber risk* is rapidly increasing with [2,3,7]:

1. The so-called dematerialization of administrative processes;
2. The increased dependence on *computerized biomedical* and *non-biomedical technologies* (as described above);
3. The large amount of data stored in the Hospital Information Systems (HIS).

Recently, we have assisted in attacks on the HIS, both based on viruses (in minor cases) and by real complex systems, managed by increasingly capable and ingenious unlawful organizations. This means that the HIS can be attacked and breached in terms of both privacy and of activities [7]. It should be considered that the HISs have a criticality of the highest level, since the activity (based on specifically designed softwares) is linked to the health of people. With regard to the HIS, *cybersecurity* has, therefore, a leading role in the defense of IT infrastructures and in the final analysis of the citizen.

## 4. Conclusions

The *cybersecurity* in healthcare includes all the general actions employed both in the consumer and industrial sectors (Network Security, Application Security, Information Security, Operational Security, Disaster Recovery and Operational Continuity, End-User Training) tuned and specialized for the health-care sector. It should be considered that the criticality of the healthcare systems is of the highest level, since the activity is linked to the health of people; therefore, a correct and effective implementation of the cybersecurity assumes the utmost importance. All traditional health sectors and those emerging from eHealth and mHealth must be addressed with the utmost attention, and can and should be investigated by scholars. Training and information must be key aspects of cybersecurity in healthcare.

*It will be necessary to foresee specific investigations with targeted scientific studies in each of the above-described fields. It will be also necessary to set up specific studies based on survey tools, to understand the perception and the state of the correct use of cybersecurity on the actors involved, from the medical specialist to the common people, who are disadvantaged and have a low level of instruction.*

*It could be also useful to understand whether it is appropriate to expand and better generalize the role of cybersecurity in new border areas of the health sector, such as, for example, (a) the sector of non-medical apps that can be confused with medical devices and whose non-compliant use could put patient safety at risk, especially during this COVID-19 pandemic period (perspective articles here are also strongly needed and welcome), and (b) the sector of the new Apps for the digital contact tracing, where discussion is increasing on the position of the citizen: with, on the one hand, his or her privacy rights and, on the other hand, the need to make every effort in order to stop the Covid-19 pandemic (reviews which analyze this issue are also welcome here) [8,9].*
